# Anti-Obesity Properties of Blackberries Fermented with *L. plantarum* JBMI F5 via Suppression of Adipogenesis Signaling Mechanisms

**DOI:** 10.3390/ijms25116164

**Published:** 2024-06-03

**Authors:** Jae Young Park, Ha-Rim Kim, Seung-Hyeon Lee, Sang-Wang Lee, Hong-Sig Sin, Tae-Gyu Lim, Seon-Young Kim, Mi Hee Park

**Affiliations:** 1 Jeonju AgroBio-Materials Institute, Wonjangdong-gil 111-27, Deokjin-gu, Jeonju-si 54810, Jeonbuk State, Republic of Korea; jjay1205@jami.re.kr (J.Y.P.); poshrim@jami.re.kr (H.-R.K.); sh94@jami.re.kr (S.-H.L.); 2 Chebigen Inc., 62 Ballyong-ro, Deokjin-gu, Jeonju-si 54853, Jeonbuk State, Republic of Korea; cbg31@chebigen.com (S.-W.L.); shsdo@hanmail.net (H.-S.S.); 3 Department of Food Science & Biotechnology, Sejong University, Seoul 05006, Republic of Korea; tglim@sejong.ac.kr

**Keywords:** blackberries, *L. plantarum* JBMI F5, obesity, high fat diet, adipogenesis

## Abstract

Blackberries (*Rubus fruticosus*), which are known to include a variety of bioactive substances, have been extensively studied for their antioxidant properties. Blackberries possess multiple health beneficial effects, including anti-inflammation, anti-atherosclerosis, anti-tumor and immunomodulatory activity. However, the potential biological effects and precise molecular mechanisms of the fermented extracts remain largely unexplored. In this research, we demonstrate the effect of blackberries fermented with *Lactobacillus* for addressing obesity. We investigated the effect of blackberries fermented by *Lactobacillus* on mice fed a high-fat (60% kcal) diet for 12 weeks. Fermented blackberry administration reduced the body weight and epididymal fat caused by a high-fat diet compared to the obese group. The triglyceride and total cholesterol, which are blood lipid indicators, and the levels of leptin, which is an insulin resistance indicator, were significantly increased in the obese group but were significantly decreased in the fermented blackberries-treated group. Additionally, the expression of adipogenesis marker proteins, such as CEBPα, PPAR-γ and SREBP-1, was significantly increased in the obese group, whereas it was decreased in the fermented blackberries-treated group. These results suggest that fermented blackberries have a protective effect against high-fat-diet-induced obesity by inhibiting adipogenesis and are a potential candidate for the treatment of obesity.

## 1. Introduction

The global obesity epidemic affects over one billion people and is caused by an increase in adipose tissue mass due to the proliferation of adipocytes promoted by adipogenesis [1,2]. Obesity is associated with a variety of metabolic disorders, such as cardiovascular diseases, type 2 diabetes, non-alcoholic fatty liver disease, chronic kidney diseases and various forms of cancer [3]. The U.S. Food and Drug Administration (FDA) has approved some anti-obesity drugs, including orlistat, phentermine/topiramate, naltrexone/bupropion, liraglutide and semaglutide, to help with weight management [4]. Some medications used for obesity are associated with side effects including nausea, insomnia, constipation, gastrointestinal problems and potential cardiovascular complications [4]. The stimulant-type weight loss medications are typically prescribed for short-term use due to the potential risk of dependence and other associated side effects [5]. Therefore, many efforts are being made to discover anti-obesity food components that can decrease the accumulation of body fat, reduce the side effects related to clinical treatment and reduce the risk of obesity-related diseases [6]. Several studies have shown that natural products derived from nutritious and medicinal plants, with little or no side effects, have anti-obesity effects [7]. Extracts from functional foods often contain components including polyphenols and flavonoids, which have been shown to have the ability to reduce cholesterol associated with metabolic disorders, including obesity and high blood pressure [8].

Fermented foods contain a variety of bioactive compounds that have therapeutic effects, especially for metabolic disorders [9]. These ingredients exhibit various beneficial activities such as lipid lowering, blood pressure lowering, thrombolysis, anti-mutation, immune stimulation, and antibacterial and anti-hair loss effects [10]. Therefore, these ingredients play a role in preventing arteriosclerosis, heart disease, obesity, diabetes, senile dementia, cancer and osteoporosis and inflammation mediated by oxidative stress [11,12]. Basically, fermentation has been used for generations to preserve food and its texture by inactivating spoilage microorganisms [13]. When raw materials are fermented, various bioactive chemicals and enzymes that are not present in the raw materials are produced [14]. Therefore, fermented substances function as relatively optimal drug delivery systems by containing a mixture of safe bioactive compounds in sufficient quantities to deliver to the target site, promoting efficacy and long-term compliance [15,16]. Food matrices by fermentation can exert medicinal properties through the presence of large numbers of viable microorganisms and modified metabolites to protect the gastrointestinal tract from pathogens, excessive gastric acid and bile salts [17,18]. Moreover, fermented foods contain persistent microorganisms, mainly lactic acid bacteria and their major metabolites [19]. Probiotic and prebiotic supplementation has beneficial effects on obesity-related metabolic complications, including distorted lipid profiles [20]. However, the mechanism of action is not completely clear, so research on the mechanism of action of fermented foods and microorganisms is needed.

Blackberries, a type of fruit belonging to the Rubus genus, are rich in polyphenols, especially anthocyanins and other antioxidants, contributing to their high antioxidant activity [21]. Studies have revealed a variety of biological activities of blackberries, including protection against oxidative stress, endotoxicity, age-related neurodegenerative diseases, obesity, cancer and cardiovascular disease [22]. Blackberries also contain proanthocyanidins and ellagitannins, which are polymers of ellagic acid and contribute to their ability to reduce oxidative stress and inflammation [23]. It has been reported that, during the fermentation and digestion of blackberries, the phenolic compounds and gut metabolites significantly increased [24]. Also, in a previous study, we showed that fermented blackberries have a protective effect on skin aging against UV irradiation [25]. However, not much research has been conducted on the effects of fermented blackberries on other diseases. In this study, we showed that blackberries fermented by Lactobacillus have anti-obesity effects and we identified the molecular mechanisms related with adipogenesis to assess the potential of fermented blackberries for obesity treatment.

## 2. Results

### 2.1. Effects of Fermented Blackberries Administration on Adipogenesis and Lipid Accumulation

**Figure 1:**
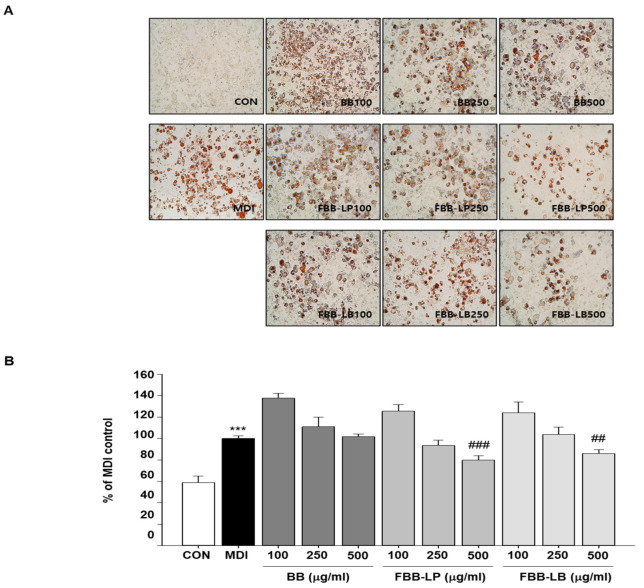
Effects of fermented blackberries on lipid accumulation in differentiating 3T3-L1 cells. (**A**) Morphological change (×40 magnification); (**B**) lipid accumulation. *** *p* < 0.001 versus CON group; ^##^ *p* < 0.01 and ^###^ *p* < 0.001 versus MDI group. BB, blackberries; FBB-LP, blackberries fermented by *L. plantarum* JBMI F5; FBB-LB, blackberries fermented by *L. brevis* CBG-C24.

Two types of fermented blackberries were studied for the differentiation of 3T3-L1 cells. The inhibitory effect of the fermented blackberries on 3T3-L1 differentiation was investigated. Fermented blackberries were treated at concentrations of 100, 250 and 500 μg/mL and cell differentiation was induced with a mixture of methyl isobutylxanthine dexamethasone and insulin, followed by Oil Red O staining. As shown in Figure 1, the fermented blackberries inhibited 3T3-L1 cell differentiation in a concentration-dependent manner.

### 2.2. Effects of Fermented Blackberries on Expression of Adipocyte Differentiation Regulatory Proteins

**Figure 2:**
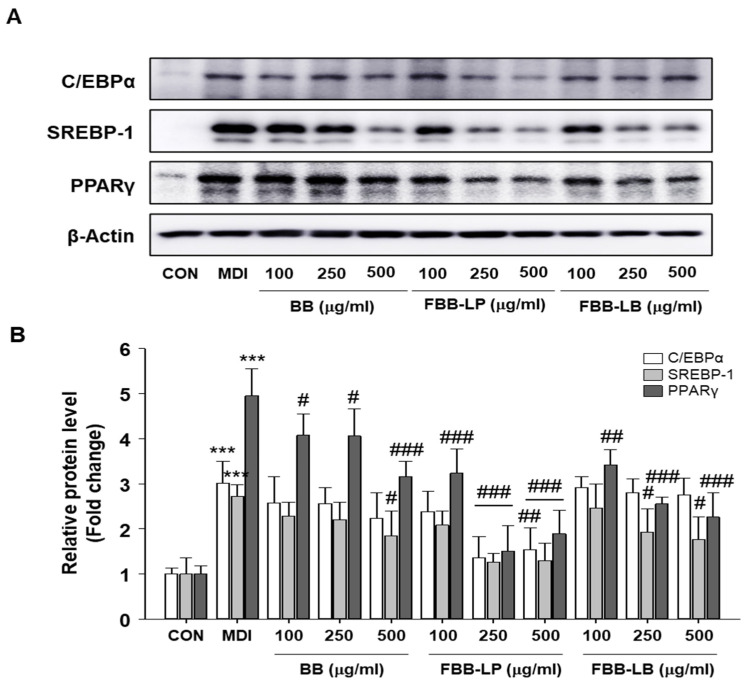
Effects of fermented blackberries on the expression of CEBP, PPAR-γ and SREBP-1 in 3T3-L1 cells. (**A**) Western blotting; (**B**) densitometry analysis. *** *p* < 0.001 versus CON group; ^#^ *p* < 0.05, ^##^ *p* < 0.01 and ^###^ *p* < 0.001 versus MDI group. BB, blackberries; FBB-LP, blackberries fermented by *L. plantarum* JBMI F5; FBB-LB, blackberries fermented by *L. brevis* CBG-C24.

To investigate adipogenesis, we showed the expression of adipocyte differentiation marker proteins, specifically CEBP, PPAR-γ and SREBP-1. The treatment of fermented blackberries effectively inhibited the expression levels of CEBP, PPAR-γ and SREBP-1 (Figure 2). The results suggest that the administration of fermented blackberries provides protection against the adipogenesis function. In particular, we showed that the treatment of fermented blackberries by *L. plantarum* (FBB-LP) dramatically reduced the expression of these adipogenesis marker proteins.

### 2.3. Effects of Fermented Blackberries on Body Weight and eWAT Weight in HFD-Fed Mice

**Figure 3:**
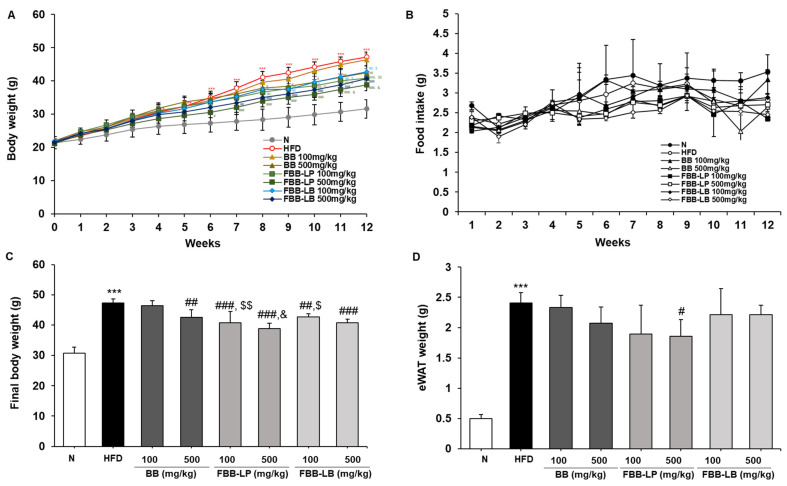
Effects of fermented blackberries on body weight and eWAT weight of HFD-induced obesity mice. (**A**) Change in body weight, (**B**) food intake, (**C**) body weight 12 weeks after inducing obesity with HFD and (**D**) eWAT weight. All values represent the mean ± SD. Data were analyzed using Turkey’s multiple comparison test. * *p* < 0.05, ** *p* < 0.01 and *** *p* < 0.001, versus the N group; ^#^ *p* < 0.05, ^##^ *p* < 0.01, and ^###^ *p* < 0.001, versus the HFD group; ^$^ *p* < 0.05 and ^$$^ *p* < 0.01, versus the BB 100 mg/kg group; ^&^ *p* < 0.05 versus the BB 500 mg/kg group; BB, blackberries; FBB-LP, blackberries fermented by *L. plantarum* JBMI F5; FBB-LB, blackberries fermented by *L. brevis* CBG-C24; eWAT, epididymal white adipose tissue; HFD, high-fat diet; N, normal.

The initial body weight was not significantly different between groups. During the four weeks of the experiment, the HFD group showed a significant increase in body weight compared to the N group. After 12 weeks, the final body weight of the HFD group increased to 47.23 ± 1.43 g, which is about 1.5 times that of the N group, while the fermented blackberries administration group had a relatively low increase in body weight (Figure 3A,C). In particular, we showed that the treatment of fermented blackberries by *L. plantarum* (FBB-LP) significantly reduced body weight compared with the blackberries (BB)-treated group. The epididymal white adipose tissue (eWAT) weight also significantly increased in the HFD group compared to the N group after 12 weeks, but the FBB-LP group showed a decrease in eWAT weight (Figure 3D).

During the experimental period, food intake did not differ significantly among the experimental groups (Figure 3B). Hence, it can be inferred that fermented blackberries, especially FBB-LP, have therapeutic potential for HFD-induced obesity.

### 2.4. Effects of Fermented Blackberries on Adipogenesis and Lipid Accumulation in HFD-Fed Mice

**Figure 4:**
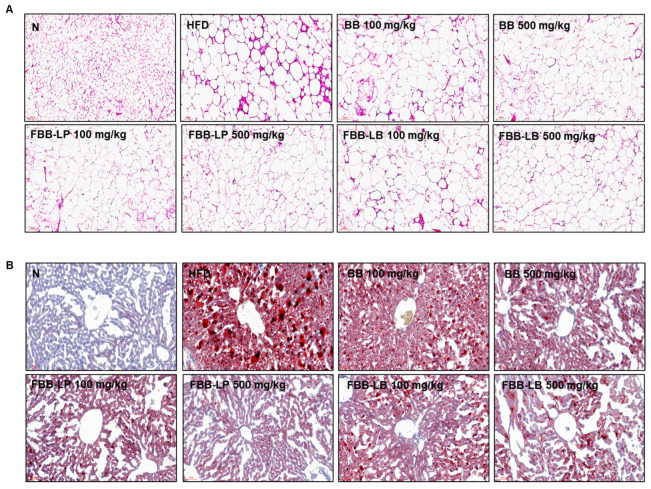
Histopathological assessment of the effects of fermented blackberries on the liver and eWAT of HFD-induced obesity mice. (**A**) Representative image of eWAT tissue stained with H&E. Magnification, 100×; scale bar, 100 μm. (**B**) Representative image of liver tissue stained with Oil Red O. Magnification, 200×; scale bar, 60 μm; BB, blackberries; FBB-LP, blackberries fermented by *L. plantarum* JBMI F5; FBB-LB, blackberries fermented by *L. brevis* CBG-C24; eWAT, epididymal white adipose tissue; HFD, high-fat diet; N, normal; H&E, hematoxylin and eosin.

To evaluate the observed changes, a histological examination was performed on WATs using H&E staining and liver tissue using Oil Red O staining. H&E staining showed a significant increase in adipocyte size in the HFD group, whereas treatment with 100 and 500 mg/kg fermented blackberries significantly decreased adipocyte size (Figure 4A). The effect of fermented blackberries on liver lipid accumulation was investigated using Oil Red O staining. The data showed that treatments with 100 and 500 mg/kg fermented blackberries reduced hepatic lipid deposition in HFD-fed mice (Figure 4B). These results demonstrated that fermented blackberry administration was effective in preventing adipocyte hypertrophy and lipid accumulation.

### 2.5. Effects of Fermented Blackberries on Hyperlipidemia in HFD-Fed Mice

**Figure 5:**
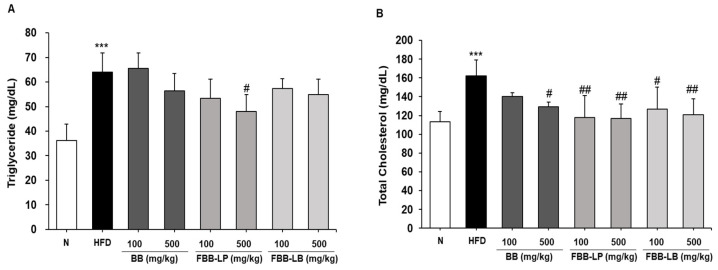
Effects of fermented blackberries on serum lipids of HFD-induced obesity mice. (**A**) TG and (**B**) TC levels. All values represent the mean ± SD. Data were analyzed using Turkey’s multiple comparison test. *** *p* < 0.001, versus the N group; ^#^ *p* < 0.05 and ^##^ *p* < 0.01, versus the HFD groups. HFD, high-fat diet; TG, triglyceride; TC, total cholesterol; BB, blackberries; FBB-LP, blackberries fermented by fermented by *L. plantarum* JBMI F5; FBB-LB, blackberries fermented by *L. brevis* CBG-C24; HFD, high-fat diet; N, normal.

The serum levels of TG and TC were measured using ELISA. The HFD significantly increased the TG and TC levels. After fermented blackberries administration for 12 weeks, the production of TG (Figure 5A) and TC (Figure 5B) decreased. In particular, we showed that the treatment with FBB-LP significantly reduced the TG contents compared with the HFD group.

### 2.6. Effects of Fermented Blackberries on Liver Toxicity in HFD-Fed Mice

**Figure 6:**
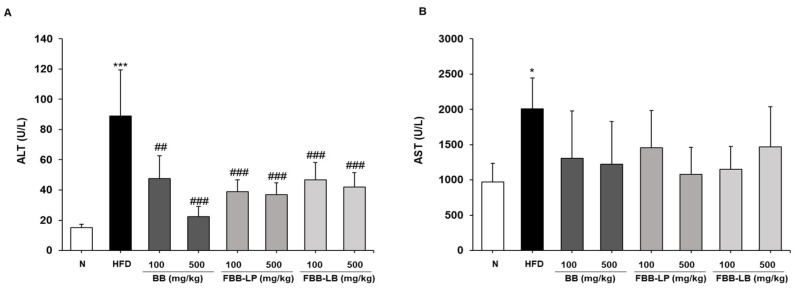
Effects of fermented blackberries on liver toxicity in HFD-induced obesity mice. (**A**) ALT and (**B**) AST levels in HFD-induced obesity mice serum. All values represent the mean ± SD. Data were analyzed using Turkey’s multiple comparison test. * *p* < 0.05 and *** *p* < 0.001, versus the N group; ^##^ *p* < 0.01 and ^###^ *p* < 0.001, versus the HFD group; BB, blackberries; FBB-LP, blackberries fermented by *L. plantarum* JBMI F5; FBB-LB, blackberries fermented by *L. brevis* CBG-C24; HFD, high-fat diet; ALT, alanine aminotransferase; AST, aspartate aminotransferase; N, normal.

To investigate whether fermented blackberries inhibit liver toxicity, serum alanine aminotransferase (ALT) and aspartate aminotransferase (AST) levels were measured. The HFD significantly increased ALT and AST levels. After treatment with fermented blackberries for 12 weeks, ALT was significantly decreased in the blackberries- and fermented blackberries-treated groups (Figure 6A), and AST was not significantly changed (Figure 6B).

### 2.7. Effects of Fermented Blackberries on HFD-Induced Insulin Resistance

**Figure 7:**
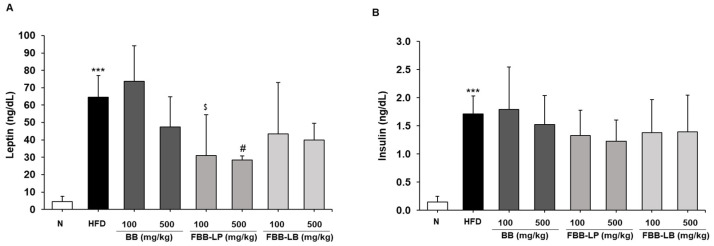
Effects of fermented blackberries on serum insulin resistance markers of HFD-induced obesity mice. (**A**) Leptin and (**B**) insulin. All values represent the mean ± SD. Data were analyzed using Turkey’s multiple comparison test. *** *p* < 0.001, versus the N group; ^#^ *p* < 0.05, versus the HFD group; ^$^ *p* < 0.05, versus the BB 100 mg/kg group; BB, blackberries; FBB-LP, blackberries fermented by *L. plantarum* JBMI F5; FBB-LB, blackberries fermented by *L. brevis* CBG-C24; HFD, high-fat diet; N, normal.

To determine whether fermented blackberries are effective against insulin resistance, leptin and insulin levels in serum were measured using ELISA. A HFD significantly increased leptin (Figure 7A) and insulin (Figure 7B) levels. The administration of 500 mg/kg fermented blackberries for 12 weeks significantly reduced leptin production.

### 2.8. Effects of Fermented Blackberries on Adipocyte Differentiation in HFD-Fed Mice

**Figure 8:**
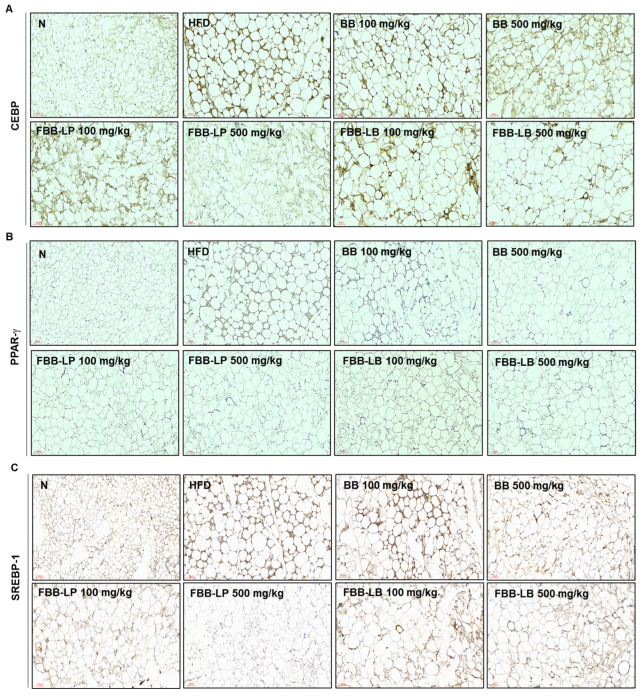
Effects of fermented blackberries on the expression of CEBP, PPAR-γ and SREBP-1 in HFD-induced obesity mice. Immunohistochemical expression patterns of the adipogenesis-related proteins including (**A**) CEBP, (**B**) PPAR-γ and (**C**) SREBP-1 in adipose tissue. Magnification: 200×, scale bar: 100 μm. BB, blackberries; FBB-LP, blackberries fermented by *L. plantarum* JBMI F5; FBB-LB, blackberries fermented by *L. brevis* CBG-C24; HFD, high-fat diet; N, normal.

To assess adipogenesis, immunohistochemistry (IHC) was performed to evaluate the expression of adipocyte differentiation marker proteins, especially CEBP, PPAR-γ and SREBP-1. CEBP, PPAR-γ and SREBP-1 exhibited uniform expression along differentiated cells in adipose tissues in the HFD group. On the other hand, the administration of fermented blackberries could effectively prevent cell differentiation and the expression levels of CEBP, PPAR-γ and SREBP-1 (Figure 8). The results suggest that the administration of fermented blackberries provides protection against adipogenic function induced by a HFD.

## 3. Discussion

This study demonstrated that fermented blackberries have an anti-obesity effect. First, we identified that fermented blackberries inhibited preadipocyte cell differentiation and lipid accumulation in a concentration-dependent manner in 3T3-L1 cells. Moreover, the expression of adipocyte differentiation marker proteins such as CEBP, PPAR-γ and SREBP-1 was dramatically decreased by fermented blackberries, suggesting that the administration of fermented blackberries provides protection against adipogenesis function. In particular, we showed that treatment with FBB-LP dramatically reduced these proteins’ expression compared with BB or fermented blackberries in the FBB-LB-treated group. Obesity is caused by the accumulation of neutral fat in adipocytes during the differentiation of 3T3-L1 preadipocytes [26]. In our in vitro study, we showed that fermented blackberries inhibited 3T3-L1 differentiation. This research showed that treatment with fermented blackberries reduced lipid droplets in differentiated 3T3-L1 cells, indicating that fermented blackberries suppress lipidogenesis and adipogenesis. Moreover, the expression of C/EBPα, PPAR-γ and SREBP-1, which are the critical factors of adipogenesis, was dramatically reduced by fermented blackberries. It has been reported that the initial factors expressed following MDI treatment are C/EBPβ and C/EBPγ [27]. During preadipocyte differentiation, C/EBPβ and C/EBPγ play a role in promoting the expression of C/EBPα and PPAR-γ, with the effect being most noticeable in the middle and late stages of differentiation [28]. In addition, a decreased expression of C/EBPα and PPAR-γ leads to the decreased expression of adipose protein 2, a pivotal factor in preadipocyte differentiation; PPAR-γ is a key regulator that is involved in adipogenesis and lipogenesis [27]. We showed that treatment with FBB-LP dramatically reduced the expression of C/EBPα, PPAR-γ and SREBP-1 compared with the BB- or FBB-LB-treated groups in 3T3-L1 cells. Therefore, our results imply that fermented blackberries can inhibit the adipogenesis of 3T3-L1 preadipocytes, and that the effect is more effective with FBB-LP treatment.

In our in vivo study, our observations revealed significant enhancements in multiple parameters linked to obesity. Remarkably, the administration of fermented blackberries significantly reduced HFD-induced body weight and eWAT weight, as well as the levels of TC and TG, indicating its potential as a viable intervention for managing obesity. The administration of fermented blackberries to HFD-induced obese mice significantly reduced the relative fat pad size. Despite the lack of effect of fermented blackberries on food intake, mice treated with fermented blackberries had lower body weight gain and lower white fat mass compared to mice that were not treated. These findings suggest that changes in caloric consumption may not always align with variations in adipose tissue mass. We also showed that fermented blackberries inhibit adipocyte differentiation and the adipogenesis regulatory proteins, including CEBP, PPAR-γ and SREBP-1. Our findings provide strong evidence for the beneficial effects of fermented blackberries on obesity. Additionally, our study demonstrated that the administration of fermented blackberries was associated with the restoration of leptin production, which is a promising result related to metabolic health.

Researchers have investigated fermented food products for their potential to exert anti-obesity effects [29]. Moreover, the fermentation process might augment the bioavailability and effectiveness of these compounds [30]. Previous research suggests that the regular consumption of fermented foods could play a role in weight control and in preventing the metabolic disorders associated with obesity [31]. In addition, fermented food products harbor probiotic bacteria, such as species of *Lactobacillus* and *Bifidobacterium*, which have the potential to modulate the composition and functionality of the gut microbiota [32]. Moreover, probiotics have been associated with reducing fat absorption, enhancing insulin sensitivity and boosting energy expenditure, all of which confer benefits in weight control and obesity prevention [33]. The bioactive constituents found in fermented food products may collaborate to regulate lipid metabolism, appetite control and gut microbiota composition, among other pathways, thereby exerting their anti-obesity effects [34]. Furthermore, adipose tissue secretes adipocytokines, such as leptin and adiponectin, with increased serum leptin and decreased serum adiponectin levels being the hallmark characteristics of obesity [35]. In another investigation, the prolonged consumption of a HFD led to elevated serum insulin levels, induced insulin resistance and facilitated enhanced fat deposition within the liver [36]. In our current research, we observed that FBB-LP significantly reduced serum leptin levels, suggesting their potential to enhance insulin clearance and notably diminish fat accumulation.

It has been reported that fermented or digested blackberries have increased phenolic compounds and gut metabolites [24]. In our previous study, we also reported that the total phenolic content, total flavonoid content and vitamin C were increased by the fermentation of blackberries [25]. Additional research is also being conducted to identify other ingredients. Actually, blackberries contain proanthocyanidins and ellagitannins, which are polymers of ellagic acid and which contribute to their ability to reduce oxidative stress and inflammation [23]. The beneficial properties of blackberries in terms of their antioxidant, anti-inflammatory and wound-healing properties have been demonstrated because of their enriched polyphenols, flavonoids and vitamins. Blackberries are rich in polyphenols, especially anthocyanins and other antioxidants, contributing to their high antioxidant activity [21]. Studies have revealed a variety of biological activities of blackberries, including protection against oxidative stress, endotoxicity, age-related neurodegenerative diseases, obesity, cancer and cardiovascular disease [22]. Also, in our previous study, we showed that fermented blackberries have a protecting effect on skin aging [25]. It has been reported that diets including fruits rich in anthocyanins may result in positive mental health outcomes [37]. Moreover, the association between obesity and depression is highly co-morbid and tends to significantly exacerbate metabolic and related depressive symptoms [38]. Therefore, controlling obesity through fermented blackberries is expected to be effective in treating mental health problems related to obesity. However, further research is needed.

In summary, our findings robustly endorse the concept that fermented blackberries exert a preventive influence on obesity and its related metabolic complexities. Additional research is necessary to clarify the underlying mechanisms related to obesity-related metabolic disorders and enhance the therapeutic effectiveness of fermented blackberries in health concerns.

## 4. Materials and Methods

### 4.1. Preparation of Fermented Blackberries

*L. plantarum* JBMI F5 and *L. brevis* CBG-C24 were grown in MRS (de Man, Rogosa and Sharpe) broth (Difco, Franklin Lakes, NJ, USA) at 37 °C for 12–16 h. The raw blackberry materials thus prepared were mixed using a crusher. The crushed blackberries were filtered using a mesh to remove seeds. The fermentation process was carried out for 24 h, while stirring at 150 rpm and 37 °C, using overnight-cultured *L. plantarum* JBMI F5 or *L. brevis* CBG-C24. After fermentation, the fermented blackberry was heated at 100 °C for 10 min and lyophilized.

### 4.2. Cell Viability

Cell viability was measured using the MTS assay. In 96-well culture plates, cells (1 × 10^4^ cells/well) were seeded and treated for 24 h with blackberries (BB), fermented blackberries by *L. plantarum* JBMI F5 (FBB-LP) and fermented blackberries by *L. brevis* CBG-C24 (FBB-LB). Then, 10 μL of MTS solution (Promega, Madison, WI, USA) was administered to the cells in each well, and incubated for an additional 4 h. At 490 nm, absorbance was determined using a microplate reader (Multiskan Go, Thermo Scientific, Waltham, MA, USA). The values of the control were considered 100% viable.

### 4.3. 3T3-L1 Preadipocytes Adipogenesis

3T3-L1 preadipocytes were purchased from ATCC (American Type Culture Collection, Manassas, VA, USA) and cultured in DMEM (Dulbecco’s Modified Eagle Medium, Gibco, Hackensack, NJ, USA) containing 10% NBCS (Newborn Calf Serum, Gibco, Hackensack, NJ, USA) and 1% of penicillin (100 U/mL)/streptomycin (100 μg/mL) at 37 °C in a 5% CO_2_ atmosphere. For differentiation, once the cells reached 100% confluence, the medium was replaced with DMEM containing MDI (0.5 mM Isobutylmethylxanthine, 1 μM dexamethasone and 10 μg/mL insulin) and 10% FBS (Fetal Bovine Serum, Thermo Scientific) for 2 days. After 2 days (D-2), the medium was switched to DMEM containing 10 μg/mL insulin and 10% FBS for an additional 2 days. Starting from day 6 (D-6) until day 8 (D-8), the cells were cultured in DMEM containing only 10% FBS. The samples were treated with the same concentrations of differentiation medium at each medium change.

### 4.4. Oil Red O Staining

To compare the effects of 3T3-L1 preadipocytes on differentiation and lipid accumulation, after the completion of differentiation, cells were fixed with 10% formalin solution and stained for lipids using an Oil Red O solution (Oil Red O:DW = 3:2). The stained Oil Red O was then extracted using 100% isopropanol, and the absorbance was assessed at 490 nm using a microplate reader (Multiskan Go, Thermo Scientific, Waltham, MA, USA).

### 4.5. Antibodies

In this study, the following antibodies were used: PPAR γ (gamma) from Invitrogen (Waltham, MA, USA); SREBP-1 from Novus Biologicals (Centennial, CO, USA); CEBP-α from Bioss Antibodies (Woburn, MA, USA); and β-actin from Cell Signaling Technology (Danvers, MA, USA).

### 4.6. Immunoblotting

Immunoblotting analysis of adipogenesis marker proteins in 3T3-L1 cells was performed following a protocol. Initially, the 3T3-L1 cells were washed with PBS; after that, they were lysed using ice-cold RIPA buffer. The lysates were then centrifuged at 10,000× *g* for 10 min and the supernatant was obtained. Equal amounts of protein (30 μg/mL) were separated by SDS-polyacrylamide gel electrophoresis and, after that, transferred to a PVDF membrane. To prevent nonspecific binding, the membranes were blocked with 5% BSA in TTBS (0.1% Tween 20 in TBS) for 1 h. The membranes were then incubated with primary antibodies (anti-C/EBPα, anti-SREBP-1, anti-PPARγ and anti-β-actin (1:2500) at 4 °C for overnight, followed by a 2 h incubation with secondary antibodies (either anti-rabbit IgG or anti-mouse IgG linked with horseradish peroxidase). Antibody-bound proteins were detected using an ECL solution, and relative protein expression was quantified using ImageJ version 1.54.

### 4.7. Animals

Four-week-old specific pathogen-free-grade male C57BL/6 mice were procured from Damul Science (Daejeon, Republic of Korea) and acclimated for 1 week. Mice were housed in a mouse cage with a 12 h light/dark cycle at 22 ± 2 °C and 55% ± 5% relative humidity. All experimental procedures were approved by the Animal Care Committee of Jeonju AgroBio-Materials Institute, Jeonju, Republic of Korea (approval number: JAMI IACUC 2023006).

### 4.8. Experimental Groups

The mice were divided into five groups: the normal group (N), the high-fat-diet-induced obesity group (HFD), the blackberry-treated group (BB) and the fermented blackberry administration group (FBB-LP, FBB-LB). To induce obesity, the HFD, BB, FBB-LP and FBB-LB groups were fed a 60% kcal fat diet for 12 weeks, and the N group was fed a normal diet (10% kcal fat). The BB, FBB-LP and FBB-LB groups were orally administered BB (100 and 500 mg/kg), FBB-LP (100 and 500 mg/kg) and FBB-LB (100 and 500 mg/kg), respectively, for 12 weeks. N and HFD groups were administered vehicle (distilled water). Five mice per group were analyzed.

### 4.9. Analysis of Biomarkers in Serum (ELISA)

Serum TG (triglyceride), TC (total cholesterol) and HDL (High-density lipoprotein) concentrations were measured using kits purchased from Asan Pharm (Seoul, Republic of Korea), and low-density lipoprotein (LDL) and insulin levels were measured using kits purchased from CrystalChem (Elk Grove, CA, USA). Leptin level was measured using kits provided by R&D systems (Minneapolis, MN, USA). Adiponectin and ALP (Alkaline Phosphatase) levels were measured using kits purchased from Mybiosource (San Diego, CA, USA). ALT (Alanine Transaminase) and AST (Aspartate Aminotransferase) levels were measured using kits purchased from Abcam (Cambridge, UK). The manufacturer’s instructions were followed for all the measurements.

### 4.10. Histological Analysis

Mouse liver and epididymal adipose tissues were fixed with 4% paraformaldehyde and embedded using paraffin. Tissue sections of 4 μm thickness were stained with Oil Red O (for liver tissues) and hematoxylin and eosin (H&E) (for adipose tissues). The sections were deparaffinized, rehydrated and incubated with antibodies overnight at 4 °C. Following this, the sections underwent treatment with an anti-rabbit Envision Plus Polymer Kit (Dako, Glostrup, Denmark) for the immunohistochemistry (IHC) analysis targeting CEBP, PPAR-γ and SREBP-1. The sections underwent hematoxylin staining and morphological characteristics were obtained using an optical microscope (Olympus, Tokyo, Japan).

### 4.11. Statistical Analyses

Data are expressed as means ± standard deviation and all statistical analyses were performed using Sigmaplot v16.0 (Systat Software Inc., San Jose, CA, USA). Statistical analysis was performed to identify differences, followed by one-way analysis of variance and Turkey’s multiple comparison test. Statistical significance was set at *p* < 0.05 difference.

## 5. Conclusions

This research underscores the robust scientific evidence demonstrating the anti-obesity potential of fermented blackberries in mice exposed to a high-fat diet. This investigation unveiled that fermented blackberries notably mitigated the increase in body weight prompted by the high-fat diet, particularly attenuating adipose tissue hypertrophy. Moreover, we identified how these effects are regulated by the inhibition of adipogenesis. Hence, fermented blackberries present promising components in functional foods aimed at reducing and managing obesity and obesity-related disorders.

## Data Availability

The data presented in this study are available in this article.
